# Ocular Drug Delivery through pHEMA-Hydrogel Contact Lenses Co-Loaded with Lipophilic Vitamins

**DOI:** 10.1038/srep34194

**Published:** 2016-09-28

**Authors:** Dasom Lee, Seungkwon Cho, Hwa Sung Park, Inchan Kwon

**Affiliations:** 1 School of Materials Science and Engineering, Gwangju Institute of Science and Technology (GIST), Gwangju 61005, Republic of Korea; 2 GEO medical Co., Ltd, Gwangju 61007, Republic of Korea; 3 Department of Biomedical Science and Engineering, Gwangju Institute of Science and Technology (GIST), Gwangju 61005, Republic of Korea

## Abstract

Ocular drug delivery through hydrogel contact lenses has great potential for the treatment of ocular diseases. Previous studies showed that the loading of lipophilic vitamin E to silicone-hydrogel contact lenses was beneficial in ocular drug delivery. We hypothesized that vitamin E loading to another type of popular hydrogel contact lenses, pHEMA-hydrogel contact lenses, improves ocular drug delivery by increasing the drug loading or the duration of drug release. Loading of vitamin E to pHEMA-hydrogel contact lenses significantly increased the loading of a hydrophilic drug surrogate (Alexa Fluor 488 dye) and two hydrophilic glaucoma drugs (timolol and brimonidine) to the lenses by 37.5%, 19.1%, and 18.7%, respectively. However, the release duration time was not significantly altered. Next, we hypothesized that the lipophilic nature of vitamin E attributes to the enhanced drug loading. Therefore, we investigated the effects of co-loading of another lipophilic vitamin, vitamin A, on drug surrogate delivery. We found out that vitamin A loading also increased the loading of the drug surrogate to pHEMA-hydrogel contact lenses by 30.3%. Similar to vitamin E loading, vitamin A loading did not significantly alter the release duration time of the drug or drug surrogate.

Contact lenses have been widely used to correct vision by directly placing them on the outer surface of the eyes^1^. Beginning recently, contact lenses have also been used for therapeutic purposes. Ocular drug delivery to the eyes through contact lenses has received much attention. Assuming the correlation of drug concentration at an intended site of action to pharmacological effects, drug delivery through contact lenses would provide a better control of drug concentration at the eyes^2^,^3^. Popular contact lenses made up of silicone or poly (2-hydroxyethyl methacrylate) (pHEMA) have been investigated for ocular delivery of several drugs, including timolol, betaxolol, epinephrine, and latanoprost^4^,^5^.

In order to achieve an effective drug delivery system using contact lenses, several technical issues, such as short drug release time and limited drug loading, should be addressed. Researchers explored various strategies including vitamin loading, molecular imprinting, and nanoparticle embedding, to overcome the hurdles^4^,^6^,^7^,^8^,^9^. In particular, for silicone-based contact lenses, co-loading of vitamin E (Fig. 1) was investigated to improve drug delivery effectiveness^10^,^11^. Vitamin E usually indicates various forms of lipophilic tocopherols. Chauhan and his colleagues revealed that vitamin E-loaded silicone contact lenses exhibited a significantly enhanced drug release duration time or drug loading for several ocular drugs, including timolol, fluconazole, and dexamethasone compared to intact contact lenses^10^,^11^. Although its mechanism was not yet completely understood, the researchers hypothesized that lipophilic vitamin E forms very small lipophilic aggregates, distributed throughout the contact lenses, which enhance retention of lipophilic drugs or hinder passage of hydrophilic drugs^12^. Despite favorable features of vitamin E for ocular drug delivery, its use in another popular kind of contact lenses, pHEMA-based contact lenses, was not yet extensively investigated. Therefore, in this study, we first evaluated the effects of vitamin E loading to pHEMA-based contact lenses on the drug loading and drug release duration time. Prior to testing hydrophilic ocular drugs, as a drug surrogate, Alexa Fluor 488 dye (Fig. 1) was examined. Alexa Fluor 488 dye was chosen as a drug surrogate. Since the dye is hydrophilic and has multiple ring structures, it was used as a surrogate of hydrophilic drugs, such as timolol and brimonidine used in this study (Fig. 1). Furthermore, the dye has good spectral properties (absorption and fluorescence) facilitating measurement of the amount of dye released. The dye is hydrophilic and has multiple ring structures. Then, two glaucoma drugs (timolol and brimonidine) were tested for drug loading and drug release time. Timolol is a beta-blocker drug, while brimonidine is an alpha-2 agonist. In particular, brimonidine was recently approved by the Food and Drug Administration^13^ and so requires more studies on ocular delivery through contact lenses.

Assuming the effects of vitamin E on ocular drug delivery are mainly attributed to its lipophilic nature, we hypothesized that another lipophilic molecule would exhibit favorable effects on ocular drug delivery similar to vitamin E. In order to test this hypothesis, we chose vitamin A as a potential drug loading enhancer considering its great biocompatibility as well as lipophilicity. Vitamin A (Fig. 1) is a group of lipophilic retinoids, including retinol, retinal, and retinyl esters^14^,^15^,^16^. Therefore, an ocular drug delivery system using vitamin A loaded contact lenses might provide additional beneficial effects on eye health. In this study, we also investigated the effects of vitamin A on the ocular delivery of a drug surrogate through pHEMA-based contact lenses.

## Materials and Methods

### Materials

High-purity 2-hydroxy ethyl methacrylate (HEMA), ethylene glycol dimethacrylate (EGD), glycidyl methacrylate (GMA), N-vinyl-2-pyrrolidone (NVP), azobisisobutyronitrile (AIBN), and contact lens molds were kindly provided by GEOmedical, Inc. (Gwangju, Republic of Korea). Alexa Fluor 488 was purchased from Life Technologies (Grand Island, NY). Brimonidine tartrate was obtained from Santa Cruz Biotechnology, Inc. (Santa Cruz, CA). Vitamin E (α-tocopherol), vitamin A (retinol), timolol maleate, and ethanol were purchased from Sigma-Aldrich (St. Louis, MO). All chemicals were used without further purification unless otherwise indicated.

### Synthesis of pHEMA based hydrogel contact lenses

The pHEMA-based hydrogel contact lenses used in this study were synthesized according to the procedures of reusable soft contact lens production by GEOmedical, Inc. The monomer solution consisted of 92% backbone monomer (HEMA) and 8% combinations of other functional co-monomers (EGD, GMA, and NVP). After the addition of AIBN, the solution was mixed well and pipetted into contact lens molds. Then, the molds were placed in an oven and cured for 1 hr at 70 °C, followed by 1 hr at 110 °C. After polymerization, the lenses were immersed in deionized water for one week in order to remove the molds and unreacted residues. The synthesized hydrogels were dried at room temperature.

### Vitamin Loading into Contact Lenses

A lens was immersed in 1.5 mL of vitamin-ethanol solution for 24 hrs at an appropriate vitamin concentration, ranging from 0 to 0.28 M. The experiments were performed in triplicate. After the loading step, the lenses were taken out and washed with ethanol and deionized water to remove excess vitamin from the surface. The samples were then dried at room temperature overnight.

### Measurement of Water Content and Size

The water content was calculated as follows:


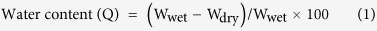


In Equation (1), W_wet_ and W_dry_ are the mass of a fully hydrated lens and the mass of a dry lens, respectively. Size of lenses were measured by a ruler.

### Loading of Drug (or Drug surrogate)

Drug (or dye) and vitamin were loaded together into a lens by soaking a lens in 1.5 mL ethanol solution containing 0.1 mg Alexa Fluor 488 dye or 0.5 mg timolol or brimonidine with vitamin for 24 hrs. Since vitamins E and A are lipophilic, they are almost insoluble in PBS buffer. Therefore, we dissolved vitamins E and A in ethanol for loading to contact lenses as reported previously^11^,^17^.The drug or dye was directly added into the 1.5 mL vitamin-ethanol solution to load vitamin and drug simultaneously (simultaneous loading). Alternatively, drug or dye was loaded into vitamin-loaded lenses by soaking the vitamin-loaded lens in a drug or dye-PBS solution. Each vitamin-loaded lens was immersed in 1.5 mL PBS solution containing 0.1 mg Alexa fluor 488, 0.5 mg timolol, or 0.5 mg brimonidine for 24 hrs (sequential loading). At the end of the loading step, the lenses were taken out and washed with ethanol and deionized water to remove excess drug or dye from the surface. The lenses were then dried at room temperature overnight in order to remove residual ethanol prior to drug release tests.

### *In Vitro* Drug Release Tests

The timolol and brimonidine (and Alexa Fluor 488 dye) release tests were carried out. Each drug- (or dye-) loaded lens was soaked in 3 mL fresh PBS buffer (pH = 7.4). During the release tests, the released drug (or dye) concentration in the PBS buffer was determined every 30 minutes by measuring the absorbance of solution at 495 nm for Alexa Fluor 488 dye, 294 nm for timolol, and 249 nm for brimonidine with a Synergy H1 four multimode microplate reader (BioTek, Winooski, VT).

## Results and Discussion

### Preparation of Vitamin-Loaded pHEMA-Hydrogel Contact Lenses

pHEMA-hydrogel contact lenses were prepared by polymerizing 2-hydroxy ethyl methacrylate (HEMA) monomer in the presence of ethylene glycol dimethacrylate (EGD), glycidyl methacrylate (GMA), N-vinyl-2-pyrrolidone (NVP), and azobisisobutyronitrile (AIBN). Both vitamin E (α-tocopherol) and vitamin A (retinol) were completely dissolved in ethanol in an appropriate concentration. Then, dry pHEMA-hydrogel contact lenses were immersed in the vitamin solution for 24 hrs in order to load the vitamin. After washing, two important physical properties (water content and size) of vitamin-loaded contact lenses were compared with those of intact contact lenses. Neither vitamin E loading nor vitamin A loading significantly changed the water content or size of the contact lenses (p > 0.05; Table 1). The vitamin-loaded contact lenses were subjected to drug release tests.

### Release Test of a Drug Surrogate Using Vitamin E-Loaded pHEMA-Hydrogel Contact Lenses

**Figure 2:**
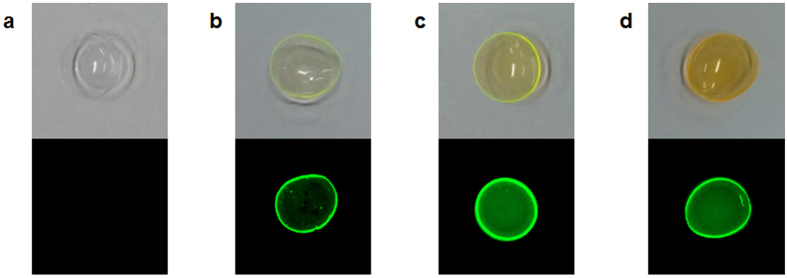
White-light (*top* panels) and fluorescence (*bottom* panels) images of contact lenses without a drug surrogate (Alexa Fluor 488) (**a**), loaded with a drug surrogate alone (**b**), and co-loaded with a drug surrogate and vitamin E (**c**) or vitamin A (**d**).

As a drug surrogate, Alexa Fluor 488 dye was used to examine whether vitamin E loading to pHEMA-hydrogel contact lenses provides beneficial effects on ocular drug delivery. In order to load Alexa Fluor 488 dye and vitamins to the lenses, the lenses were immersed in ethanol solution containing Alexa Fluor 488 only or Alexa Fluor 488 plus vitamin E for 24 hrs. Using the imager (ChemiDoc XRS imager, Bio-Rad), white-light and fluorescence images of the lenses were taken. The lenses without the dye did not exhibit any color or fluorescence (Fig. 2a). However, all lenses immersed in ethanol solution containing the dye showed a yellowish color (*top* panels in Fig. 2b,c) or fluorescence (*bottom* panels in Fig. 2b,c). Vitamin E co-loaded lenses exhibited stronger fluorescence compared with vitamin-free lenses (*bottom* panels in Fig. 2b,c), indicating that co-loaded vitamin E increased the amount of dye loaded.

**Figure 3:**
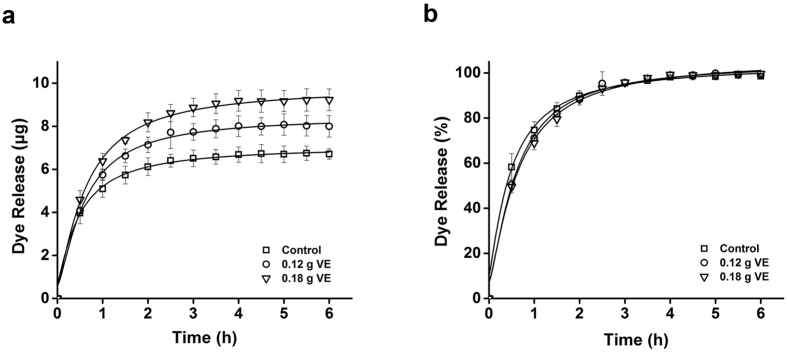
Profiles of Alexa Fluor488 dye released from pHEMA-hydrogel contact lenses co-loaded with vitamin E. Y-axis indicates the amount of dye released (**a**) and % of the dye released (**b**). The lenses were immersed in 1.5 mL ethanol solution containing 0.1 mg of the dye without vitamin E (Control) or with 0.12 g, or 0.18 g of vitamin E (VE), respectively. All measurements were performed in triplicate. Error bars indicate standard deviations.

The lenses containing the dye were subjected to release tests. The lenses were placed in fresh PBS buffer for an appropriate time period. As shown in Fig. 3a, the amount of dye released from the vitamin E loaded lenses was significantly greater than that from the intact lenses by up to 37.5% (p < 0.05). Since the amount of dye released reached plateau, the increased amount of dye released was attributed to the increased amount of dye loaded to the lenses. Therefore, in this study, we assumed that the total amount of dye or drug released directly correlated to the total amount of dye loaded. The release test results were in agreement with those obtained from fluorescence image analysis of the lenses (Fig. 2). In case of immersing lenses in the vitamin E-ethanol solution, the amount of vitamin E loaded to the lenses is directly proportional to the concentration of vitamin E in ethanol^11^,^13^. Therefore, the release test results of the dye suggested that the increase in amount of dye loaded is proportional to the amount of vitamin E loaded.

Since vitamin E significantly enhanced the release duration time in silicone-hydrogel contact lenses, we also compared the release duration time of the lenses with and without vitamin E (Fig. 3b). However, there was no significant difference in the presence of vitamin E. Although the exact mechanism was not revealed to explain the enhanced release duration time in the vitamin E loaded silicone-hydrogel contact lenses, we speculated that, at least, the effects of vitamin E on drug delivery by pHEMA-hydrogel contact lenses were different from those on drug delivery by silicone-hydrogel contact lenses^11^,^17^.

### Release Test of Drugs Using Vitamin E-Loaded pHEMA-Hydrogel Contact Lenses

**Figure 4:**
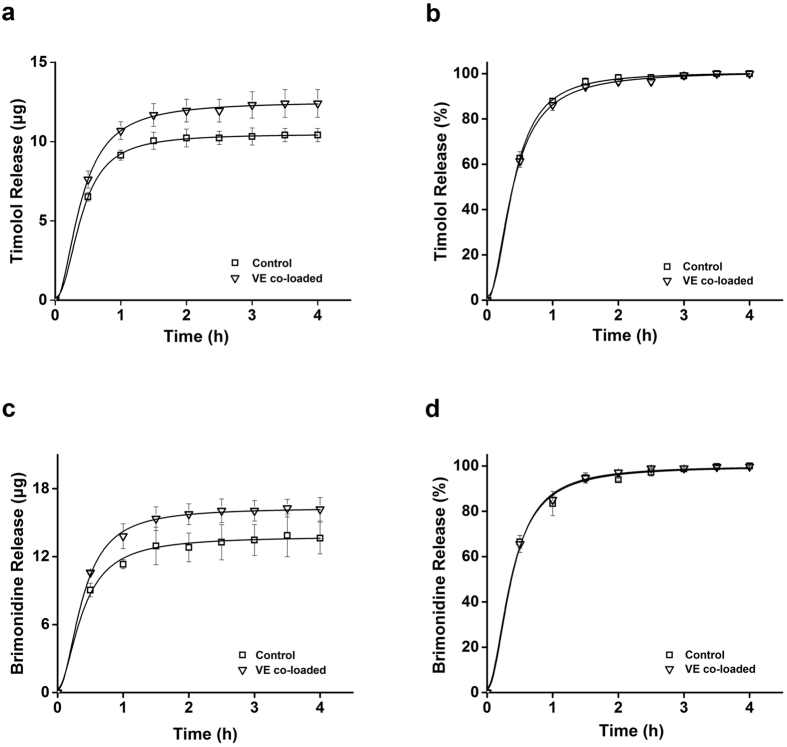
Profiles of ocular drugs released from pHEMA-hydrogel contact lenses co-loaded with vitamin E. Y-axis indicates the amount of timolol (**a**) or brimonidine (**c**) released, and % of timolol (**b**) or brimonidine (**d**) released. The lenses were immersed in 1.5 mL ethanol solution containing 0.5 mg of the drug without vitamin E (Control) or with 0.18 g of vitamin E (VE), respectively. All measurements were performed in triplicate. Error bars indicate standard deviations.

Similarly to the dye release tests, we performed the release tests of two hydrophilic ocular drugs, timolol and brimonidine, using vitamin E loaded pHEMA-hydrogel contact lenses, except 0.5 mg of the drugs instead of 0.1 mg of the dye in ethanol solution. Since these drugs do not have fluorescence properties, fluorescence image analysis of the lenses was not performed. With results similar to those of the dye release tests, vitamin E loaded contact lenses significantly increased the total amount of timolol and brimonidine released by 19.1% and 18.7%, respectively (p < 0.05) (Fig. 4a,c). These results also suggested that co-loading of vitamin E increased the amount of the drugs loaded. Consistently with the dye release test results, the release duration times of the drugs were not significantly changed (Fig. 4b,d).

In case of silicone-hydrogel contact lenses, vitamin E loading increased the total amount of timolol released by up to about 20%^11^. Vitamin E loading to the silicone-hydrogel contact lenses enhanced the release duration time of timolol more than ten times compared to intact lenses^11^. The authors hypothesized that lipophilic vitamin E in the silicone contact lenses form aggregates acting diffusion barrier for the hydrophilic timolol. We also speculated that aggregate formation of vitamin E inside pHEMA contact lenses led to the increase in the amount of timolol loaded. However, considering no change in the release duration time of timolol in vitamin E-loaded pHEMA contact lenses, it was not very likely that vitamin E acted diffusion barrier for timolol in pHEMA contact lenses. We speculated the hydrophilic nature of pHEMA contact lenses resulted in the different role of vitamin E compared to the hydrophobic silicone contact lenses.

### Release Test of a Drug Surrogate Using Vitamin A Loaded pHEMA-Hydrogel Contact Lenses

**Figure 5:**
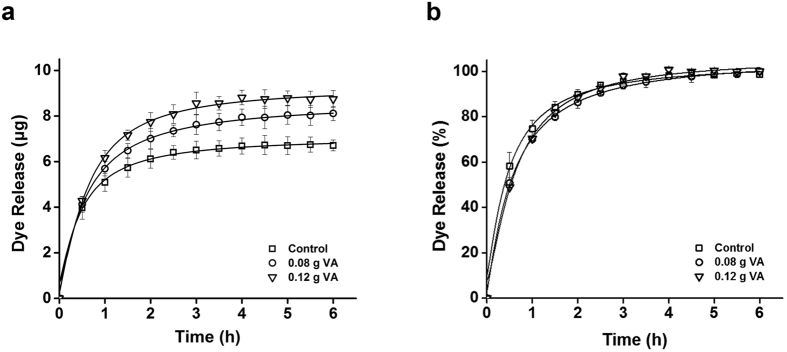
Profiles of Alexa Fluor488 dye released from pHEMA-hydrogel contact lenses co-loaded with vitamin A. Y-axis indicates the amount of dye released (**a**) and % of the dye released (**b**). The lenses were immersed in 1.5 mL ethanol solution containing 0.1 mg of the dye without vitamin E (Control) or with 0.08 g, or 0.12 g of vitamin A (VA), respectively. All measurements were performed in triplicate. Error bars indicate standard deviations.

Release tests of the drug surrogate (Alexa Fluor488 dye) using vitamin A loaded pHEMA-hydrogel contact lenses were performed under similar conditions, except with vitamin A instead of vitamin E in ethanol solution. In order to load the dye and vitamin A, the lenses were immersed in ethanol solution containing Alexa Fluor 488 only or Alexa Fluor 488 plus vitamin A for 24 hrs. Using the imager, white-light and fluorescence images of the lenses were taken. The vitamin A loaded contact lenses containing the dye showed a yellowish color (*top* panel in Fig. 2d) or fluorescence (*bottom* panel in Fig. 2d). Vitamin A co-loaded lenses showed stronger fluorescence compared with vitamin-free lenses (*bottom* panels in Fig. 2b,d), indicating that co-loaded vitamin A increased the amount of dye loaded. As expected, the vitamin A loaded lenses released about 30.3% more dye compared with the vitamin-free contact lenses (p < 0.05) (Fig. 5a). Similarly, the release duration time was not changed even with co-loading of vitamin A. We could not perform the timolol or brimonidine release tests on vitamin A loaded contact lenses, due to spectral interference. In order to estimate the amount of drugs released, we measured absorbance of timolol at 294 nm and brimonidine at 249 nm. Since vitamin E loaded to the lenses was not significantly released during release tests, it was straightforward to estimate the amount of drugs from absorbance measured. However, vitamin A was also released along with the dye during the release test and exhibited strong absorbance at 294 nm and 249 nm, hindering accurate measurement of drugs based on absorbance. In the future, in order to estimate accurate concentrations of drugs released from vitamin A loaded lenses, drugs and vitamin A should be separated prior to absorbance measurement, using high performance liquid chromatography, for example.

**Figure 6:**
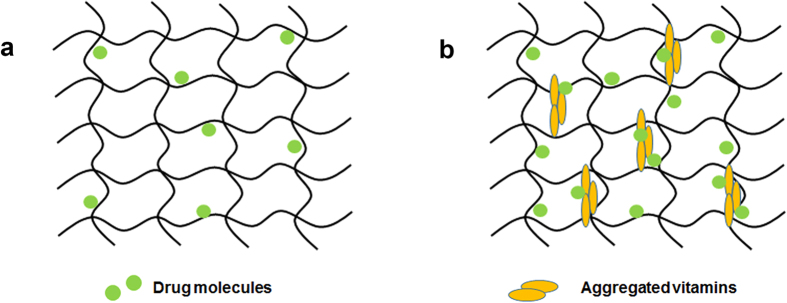
Schemes of drug-loaded hydrogel network (**a**) and drug-vitamin co-loaded hydrogel network (**b**).

All results suggested that co-loading of lipophilic vitamin E or vitamin A increased the amount of a drug or drug surrogate loading to pHEMA-hydrogel contact lenses, but did not alter the release duration time. Considering that both vitamins are non-polar but pHEMA-hydrogel is highly polar, we speculated that additional drug loading around vitamin aggregates is attributed to the increased amount of drug loaded. Vitamin E aggregate formation in silicone-hydrogel contact lenses was also proposed previously^11^,^12^,^17^. Since vitamins are not soluble in water, they were dissolved in ethanol for loading of the dye/drug and vitamin. Although the dye and drugs do not have any charge in ethanol, it was expected that there would be weak favorable interactions between the dye/drug and vitamin in ethanol solution. During the drying step, we speculated that non-polar vitamins formed aggregates inside the highly polar hydrogel network, and then some dyes/drugs are additionally placed around the vitamin aggregates, leading to the increase in the amount of dye/drug loaded (Fig. 6).

**Figure 7:**
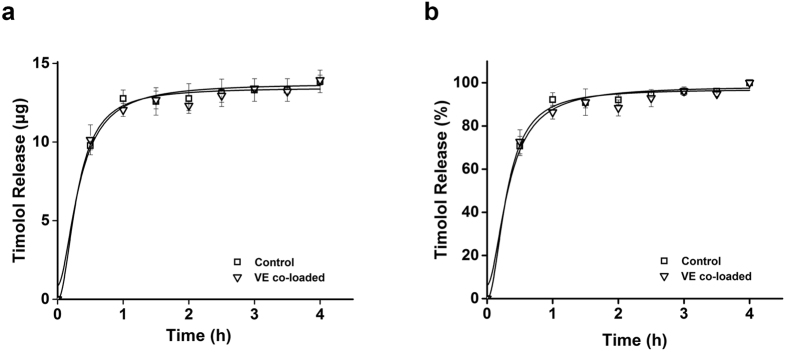
Profiles of timolol released from pHEMA-hydrogel contact lenses loaded with vitamin E. Y-axis indicates the amount of timolol released (**a**) and % of timolol released (**b**). The lenses were immersed in 1.5 mL ethanol solution containing vitamin E (VE). After washing, the lenses were immersed in 1.5 mL PBS buffer containing timolol. All measurements were performed in triplicate. Error bars indicate standard deviations.

During the release tests, the lenses were placed in PBS buffer. In PBS buffer, the dye/drugs have charges and so are easily detached from the vitamin aggregates, explaining why there was no significant increase in the release duration times. In order to test this hypothesis, we loaded timolol into vitamin E-loaded hydrogel contact lenses in PBS buffer and performed release tests. According to the hypothesis described above, vitamin E favorably interacts with timolol in ethanol, but not in PBS buffer. According to the release test results shown in Fig. 7, as expected, loading of vitamin E into pHEMA-hydrogel contact lenses did not significantly alter release profiles. These results support our hypothesis as favorable interactions of vitamins with drug or dye in ethanol as cause for the enhanced loading of drug or dye.

Since the final amount of drug loading was not dependent of time, we considered it an equilibrium property rather than a kinetic one. One of key characteristics of hydrogel is the equilibrium partition coefficient, *k*_*i*_, of a dilute solute *i* defined by


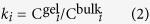


In Equation (2), C^gel^_*i*_ is the solute *i* concentration in the hydrogel per unit volume of swollen hydrogel and C^bulk^_*i*_ is the solute *i* concentration in the bulk solution equilibrated with hydrogel^18^. In case of simultaneous loading of vitamin E and a drug, the solvent for a drug (solute *i*) was ethanol. Since C^bulk^_*i*_ was the same regardless of vitamin, the difference in the amount of a drug loaded could be attributed to the difference in C^gel^_*i*_. In ethanol, timolol does not have any charge. Therefore, in the presence of vitamin, there is likely some partitioning of timolol into vitamin E in ethanol leading to the enhanced C^gel^_*i*_ compared to C^gel^_*i*_ in the absence of vitamin.

## Conclusion

Co-loading of lipophilic vitamin E to pHEMA-hydrogel contact lenses significantly increased the loading of a hydrophilic drug surrogate (Alexa Fluor 488 dye) and two hydrophilic glaucoma drugs (timolol and brimonidine) by 37.5%, 19.1%, and 18.7%, respectively. According to our hypothesis of vitamin E lipophilicity as a main cause of the enhanced drug loading, we also evaluated the effects of co-loading of lipophilic vitamin A to the lenses on loading and release time of the drug surrogate. Similarly to vitamin E, co-loading of vitamin A significantly increased the loading of the drug surrogate, supporting our hypothesis. Since the drugs are very expensive compared with the vitamins, the increase in drug loading will be beneficial for drug delivery through pHEMA-hydrogel contact lenses. More broadly, our findings demonstrated the concept of co-loading of other biocompatible, lipophilic molecules beyond vitamin E to improve the ocular drug delivery through pHEMA-hydrogel contact lenses.

## Additional Information

**How to cite this article**: Lee, D. *et al*. Ocular Drug Delivery through pHEMA-Hydrogel Contact Lenses Co-Loaded with Lipophilic Vitamins. *Sci. Rep.* **6**, 34194; doi: 10.1038/srep34194 (2016).

## Figures and Tables

**Figure 1:**
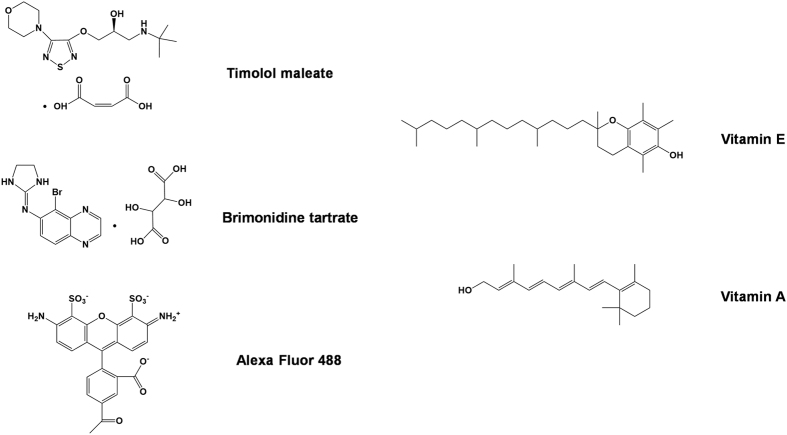
Chemical structures of Alexa Fluor 488 dye, glaucoma drugs, and vitamins used in this study.

**Table 1:** Water content^1^ and size^2^ of contact lenses.

Vitamin loaded	W_dry_ (mg)	W_wet_ (mg)	Q (%)^3^	Size (mm)
—	22.2 ± 0.2	36.0 ± 0.4	38.2 ± 0.4	14.1 ± 0.0
Vitamin E	22.0 ± 0.0	35.5 ± 0.4	37.9 ± 0.6	14.1 ± 0.1
Vitamin A	22.4 ± 0.2	36.0 ± 0.4	37.6 ± 0.6	14.1 ± 0.0

^1^Six lenses were used to obtain the average values.

^2^Four lenses were used to obtain the average values.

^3^Water content (%) = (W_wet_ − W_dry_)/W_wet_  × 100.
